# Leveraging image complexity in macro-level neural network design for medical image segmentation

**DOI:** 10.1038/s41598-022-26482-7

**Published:** 2022-12-24

**Authors:** Tariq M. Khan, Syed S. Naqvi, Erik Meijering

**Affiliations:** 1 School of Computer Science and Engineering, University of New South Wales, Sydney, NSW Australia; 2 Department of Electrical and Computer Engineering, COMSATS University, Islamabad, Pakistan

**Keywords:** Computational biology and bioinformatics, Medical research

## Abstract

Recent progress in encoder–decoder neural network architecture design has led to significant performance improvements in a wide range of medical image segmentation tasks. However, state-of-the-art networks for a given task may be too computationally demanding to run on affordable hardware, and thus users often resort to practical workarounds by modifying various macro-level design aspects. Two common examples are downsampling of the input images and reducing the network depth or size to meet computer memory constraints. In this paper, we investigate the effects of these changes on segmentation performance and show that image complexity can be used as a guideline in choosing what is best for a given dataset. We consider four statistical measures to quantify image complexity and evaluate their suitability on ten different public datasets. For the purpose of our illustrative experiments, we use DeepLabV3+ (deep large-size), M2U-Net (deep lightweight), U-Net (shallow large-size), and U-Net Lite (shallow lightweight). Our results suggest that median frequency is the best complexity measure when deciding on an acceptable input downsampling factor and using a deep versus shallow, large-size versus lightweight network. For high-complexity datasets, a lightweight network running on the original images may yield better segmentation results than a large-size network running on downsampled images, whereas the opposite may be the case for low-complexity images.

## Introduction

Medical image segmentation aims to delineate organs or lesions in images from computed tomography (CT), magnetic resonance imaging (MRI), optical imaging, and other medical imaging modalities, and serves as a basis for subsequent quantitative image analysis in a wide range of clinical and research applications. It is one of the most difficult tasks in medical image analysis, as it provides critical information about organ shapes and volumes, and medical images can be quite complex^1–4^. The challenges of obtaining a clinically applicable segmentation are multifaceted, including diverse segmentation tasks, different modalities, multiple resolutions, and varying anatomical characteristics such as shape, size, location, deformity, and texture. Recent progress in encoder-decoder architectures such as U-Net^5–8^ has improved segmentation performance on many benchmarks. However, designing such networks requires significant effort in choosing the right network configuration.

The size of medical imaging datasets is constantly increasing^9^ and often it is not possible to train deep neural network architectures on a single mid-range graphics processing unit (GPU) at the native image resolution. As a result, the images are typically downsampled before training, which may cause loss or alteration of fine details that are potentially important for diagnosis. Also, in benchmarking studies, downsampling is sometimes used for both training and testing of medical image segmentation methods^10,11^, and thus the results may not be fully representative of performance on the native images. Alternatively, shallow networks are often proposed^12–14^, in an attempt to trade off image size and network size to allow training on limited computing hardware. Another common practice is iterative downsampling until training of a deeper network of choice becomes feasible on given hardware. While these approaches are understandable from a practical standpoint, we argue that the optimal choice of input size and network depth is inherently dependent upon the characteristics of the data and the segmentation task.

Recent methods in medical image segmentation adopt neural architecture search (NAS)^15–20^ to determine the best suitable network architecture for the task at hand. However, a computationally expensive search has to be performed for each new dataset and task, and the resulting architecture may not generalize well to other datasets and tasks. Here again, the importance of the information content of the data is often ignored. We argue that we need to take a step back and base the macro-level design choices of neural networks, such as the amount of downsampling or the depth of the network, on the information complexity of the data.

Our objective in this work is to employ measures of image complexity to guide macro-level neural network design for medical image segmentation. We focus specifically on balancing input image downsampling and network depth/size for optimal segmentation results. To this end, we consider four statistical complexity measures: delentropy^21^, mean frequency^22^, median frequency^22^, and perimetric complexity^23^. Delentropy and perimetric complexity have been used previously as measures of data complexity in autonomous driving^24^ and binary pattern recognition^23^, respectively, while mean and median frequency have been used in electromyography signal identification^22^. In this paper, they are used for the first time as complexity measures for predicting a suitable input image downsampling factor and selecting a shallow versus deep, lightweight versus large-size neural network.

In general, the architectural design choices for semantic segmentation networks boil down to either model scaling^25^ (in the pursuit of performance) leading to deep networks, or model compression^26^ (for embedded and edge applications) resulting in shallow counterparts. The intended applications and corresponding hardware resources impose demands and limits on the number of trainable network parameters, and determine whether to use a computationally heavy or lightweight network. Based on model scaling and model compression, four design combinations, including deep large-size, deep lightweight, shallow large-size, and shallow lightweight networks are included in our experiments (Table 1). Here, networks with more versus less than 80 layers are categorized as deep versus shallow, and networks with more versus less than 3 million parameters are categorized as large-size versus lightweight. Based on these criteria, four existing state-of-the-art networks are selected for the comparative analysis. Specifically, DeepLabV3+^27^ is used as a deep large-size network, M2U-Net^28^ as a deep lightweight network, an adapted U-Net^5^ as a shallow large-size network, and U-Net Lite as a shallow lightweight network. To find the best complexity measure in selecting a suitable network, we use several data fitting models, including linear and polynomial fitting such as linear regression $\text {R}^2$, adjusted $\text {R}^2$, root mean square error (RMSE), mean absolute error (MAE), Akaike information criterion (AIC), and corrected AIC.

**Table 1:** Selection criteria used in this study for each of the four distinct categories of networks.

Category	Criteria	Network	Layers	Parameters
Deep large-size	$>80$ layers & >3M parameters	DeepLabV3+	100	20 M
Deep lightweight	$>80$ layers & <3M parameters	M2U-Net	155	0.55 M
Shallow large-size	$<80$ layers & >3M parameters	U-Net	58	30 M
Shallow lightweight	$<80$ layers & <3M parameters	U-Net Lite	46	0.28 M

The aim of this work is to take advantage of image complexity in the design of macro-level neural networks for medical image segmentation. To demonstrate the efficacy and wide applicability of image complexity analysis for neural network based medical image segmentation, we present experiments on 10 different datasets from public challenges. The results confirm that the proposed complexity measures can indeed aid in making the said macro-level design choices and that median frequency is the best measure for this purpose. More specifically, the results show that input image size is important for datasets with high complexity and downsampling negatively affects segmentation performance in such cases, whereas downsampling does not significantly affect performance for datasets having low complexity. Also, in the case of high-complexity datasets and computational constraints, a shallow network taking the original images as input is to be preferred, whereas for low-complexity cases competitive performance with the same computational constraints is achievable by using downsampling and a deep network topology.

## Complexity measures

It has long been known that data complexity measures can be used to determine the intrinsic difficulty of a classification task on a given dataset^29^. In this study we consider four important complexity measures and investigate their suitability for medical image segmentation tasks.

### Delentropy

The standard Shannon entropy of a gray-scale image is defined as^21^:

1 $${\text{H}} = - \sum\limits_{{i = 0}}^{{N - 1}} {p_{i} } \log p_{i} ,$$

where *N* is the number of gray levels and $p_i$ is the probability of a pixel having gray level *i*. Delentropy (DE) is computed similarly, but using a probability density function known as deldensity^21^. DE is different from Shannon entropy, which looks only at individual pixel values. Instead, DE considers the underlying spatial image structure and pixel co-occurrence through the deldensity, which is based on gradient vectors in the image. Specifically, the two-dimensional probability density function (normalized joint histogram) $p_{i,j}$ is computed as:

2 $$\begin{aligned} {p_{i,j} = \frac{1}{4WH}\sum _{w=0}^{W-1}\sum _{h=0}^{H-1}\delta _{i,d_x(w,h)}\delta _{j,d_y(w,h)},} \end{aligned}$$

where $d_x$ and $d_y$ denote the derivative kernels in the *x* and *y* direction, $\delta$ is the Kronecker delta to describe the binning process in histogram generation, and *W* and *H* are the image width and height, respectively. From this, DE is computed as:

3 $$\begin{aligned} {\text {DE} = - \frac{1}{2} \sum _{j=0}^{J-1}\sum _{i=0}^{I-1} p_{i,j} \log _2 p_{i,j},} \end{aligned}$$

where *I* and *J* are the number of bins (discrete cells) in the two dimensions of the probability density function. The $\frac{1}{2}$ factor in (3) reflects the Papoulis generalized sampling, which halves the entropy rate^21^. Discrete 2$\times$2 kernels are used as $d_x$ and $d_y$ in our implementation to estimate the *x* and *y* derivatives by taking finite differences.

### Mean frequency

The mean frequency (MNF) of a signal is computed as the sum of the product of the power spectrum and frequency divided by the total sum of the power spectrum^22^:

4 $$\begin{aligned} \text {MNF} = \frac{\sum \limits _{i=1}^{M} f_i P_i}{\sum \limits _{i=1}^{M} P_i}, \end{aligned}$$

where $P_i$ is the value of the power spectrum at frequency bin *i*, $f_i$ is the actual frequency of that bin, and *M* is the total number of frequency bins. The power spectrum is computed as the squared amplitude of the Fourier transform. Prior to power spectrum estimation, the image is windowed with a rectangular window of length determined by the dimensions of the image. The MNF can be considered as the frequency centroid or the spectral center of gravity and is also called the mean power frequency and mean spectral frequency in several works^22^. For an extension to the 2D image domain, the 1D formula (4) is first applied to each column of the image independently to obtain its mean frequency, and subsequently to the resulting vector of mean frequencies.

### Median frequency

The median frequency (MDF) of a signal is the frequency at which the power spectrum of the signal is divided into two regions with equal integrated power^22^. In other words, at the $\text {MDF}=f_j$ the following equality holds:

5 $$\begin{aligned} \sum \limits _{i=1}^{j} P_i = \sum \limits _{i=j}^{M} P_i. \end{aligned}$$

Similar to MNF, the MDF of a 2D image is computed by first applying the 1D procedure to each column independently, and then to the resulting vector. The power within each bin is computed by rectangular integration. Afterwards, the MDF is determined by searching for the bin *j* that satisfies the condition (5).

### Perimetric complexity

The perimetric complexity (PC) is a measure of the complexity of binary images. The general concept goes back to the early days of vision research^23^ where this measure, originally called dispersion, was used to describe the perceptual complexity of visual shapes. It is defined as:

6 $$\begin{aligned} \text {PC} = \frac{P^2}{4\pi A}, \end{aligned}$$

where *P* represents the perimeter of the foreground and *A* is the foreground area. In our study, this measure is computed from the annotation masks of the gray-scale images.

## Segmentation networks

To investigate the interplay between image complexity, input downsampling, and network depth and size, we considered four possible network design options: deep large-size (DeepLabV3+), deep lightweight (M2UNet), shallow large-size (U-Net), and shallow lightweight (U-Net Lite).

### Deep large-size network

DeepLabV3+^27^ was used as a deep large-size network. Consisting of 100 layers and 20 million trainable parameters, it enhances DeepLabV3 by including a simple yet effective decoder module to refine segmentation results, particularly along object boundaries^27^. We built a DeepLabV3+ network using ResNet-18 as the base network.

### Deep lightweight network

M2U-Net^28^ was employed as a representative a deep lightweight network. It uses a new encoder-decoder architecture based on the U-Net and consists of 155 layers and 0.55 million trainable parameters. Specifically, it incorporates MobileNetV2^30^ pretrained components in the encoder and novel contractive bottleneck blocks in the decoder, which, when combined with bilinear upsampling, drastically reduces the parameter count to 0.55 million compared to about 30 million in the original U-Net^5^.

### Shallow large-size network

The U-Net^5^ architecture was adopted as a shallow large-size network. It is made up of two subnetworks, namely an encoder and a decoder, which are linked by a bridge section. The encoder and decoder subnetworks are divided into several stages, the number of which determines the depth of the subnetworks. In our experiments, the encoder depth was set to 4 stages to make the U-Net a shallow network, totalling 58 layers and about 30 million trainable parameters. The U-Net encoder stages consist of two sets of convolutional and rectified linear unit (ReLU) layers, followed by a 2-by-2 max pooling layer. The decoder stages consist of an upsampling transposed convolutional layer followed by two sets of convolutional and ReLU layers. For the convolutional layers, we used feature map depths of 64, 128, 256, 512 for the four stages, respectively, and 1024 for the bridge section.

### Shallow lightweight network

For a shallow lightweight network we designed U-Net Lite based on the U-Net architecture. In U-Net Lite, we reduced the encoder depth of U-Net to 3 stages. We also used a reduced number of convolutional filters in each stage to, respectively, 8, 16, and 32. Together, these modifications reduced the number of layers to 46 and the number of trainable parameters to only 0.28 million.

## Experimental results

Two experiments were performed to test the hypothesis that image complexity can and should be taken into account in making macro-level neural network design choices for medical image segmentation. In the following sections we present the network training approach, the used public datasets, segmentation performance metrics, regression analysis performance metrics, and the results of the two experiments.

### Network training

All experiments were carried out on an Intel(R) Core(TM) i7-8700 CPU with 64 GB RAM and a relatively low/mid-range GeForce GTX1080Ti GPU. Network training was done with adaptive moment estimation (Adam) and a fixed learning rate of 1e-3. After initial experimentation, the maximum number of epochs was set to 15 with a batch size of 8 to match the hardware constraints. Gradient clipping was employed based on the global $l_2$-norm with a gradient threshold of 3^31^. Weighted cross-entropy loss was used as the objective function for training all models in our experiments. To calculate the class association weights in the loss, we used median frequency balancing^32^.

### Public datasets

We used 10 publicly available datasets (Table 2) representing a range of image complexities (Table 3). We confirm that all experiments were performed in accordance with relevant guidelines and regulations.

**Table 2:** Public datasets used in the experiments.

Dataset	Organ	Number of images	Image size (Pixels)	Training	Testing
STARE	Vessel	20	700$\times$605	10	10
DRIVE	Vessel	40	584$\times$565	20	20
CHASE-DB1	Vessel	28	999$\times$960	28	8
MC	Chest	138	4020$\times$4892, 4892$\times$4020	100	38
PH2	Skin	200	768$\times$560	ISIC-2016	200
ISIC-2016	Skin	900	576-4288$\times$542-2848	900	PH2
DRISHTI-OC	Optic cup	101	2896$\times$1944	50	51
DRISHTI-OD	Optic disc	101	2896$\times$1944	50	51
PROMISE12	Prostate	274	512$\times$512	200	74
BCSS	Breast	151	1500-3000$\times$2000-4000	100	51

#### STARE

The STARE (Structured Analysis of the Retina) dataset^33^ consists of 20 color retinal fundus images acquired with a field of view (FOV) of $35^\circ$ and size 700$\times$605 pixels. There are various pathologies in 10 of the 20 images. For each of the 20 images, two expert manual segmentation maps are available of the retinal blood vessels, and we used the first of these as the ground truth. Following others^34,35^, we used 10 for training and ten for testing.

#### DRIVE

The DRIVE (Digital Retinal Images for Vessel Extraction) dataset^36^ is from a diabetic retinopathy screening program. It contains 20 color images for training and 20 for testing with a size of 584$\times$565 pixels and covers a wide age range of diabetic patients. Seven of the 40 images show small signs of mild early diabetic retinopathy. For each of the 40 images, an expert manual segmentation mask is available for use as ground truth.

#### CHASE-DB1

The CHASE-DB1 dataset^37^ (a subset of the Child Heart and Health Study in England) includes 28 color images of children. Each image is captured with a $30^\circ$ FOV centered on the optic disc and has a size of 999$\times$960 pixels. As ground truth, two different expert manual segmentation maps are available, of which we used the first for our experiments. Since there are no specific training or testing subsets, following others^11,38–40^ we used the first 20 images for training and the remaining 8 for testing.

#### MC

The Montgomery County (MC) chest X-ray dataset^41^ contains 138 frontal chest X-ray images obtained from a tuberculosis research program and is often used as a benchmark for lung segmentation. It includes 58 tuberculosis cases and 80 normal cases with a variety of abnormalities and for which expert manual segmentations are available. The images are relatively large, either $4020\times 4892$ or $4892\times 4020$ pixels. Following others^42^, we selected 100 images for training and the remaining 38 for testing.

#### PH2

The PH2 dataset^43^ (named after its provider, the Hospital Pedro Hispano in Matosinhos, Portugal) includes 200 dermoscopic images, $768\times 560$ pixels each, of melanocytic skin lesions with expert annotation to be used as ground truth in evaluating both segmentation and classification methods. Following experimental protocols of others^44–47^, we used all images in this dataset for testing, while training was done on the ISIC-2016 training images.

#### ISIC-2016

The ISIC-2016 dataset^48^ (named after the International Skin Imaging Collaboration who hosted the challenge at the 2016 IEEE International Symposium on Biomedical Imaging where this dataset was used) contains 900 dermoscopic training images of different sizes, from as small as $576\times 768$ or $718\times 542$ pixels to as large as $4288\times 2848$ pixels, with expert manual annotation for benchmarking melanoma segmentation, pattern detection, and classification methods. For testing, we used the PH2 images.

#### DRISHTI-OC

The DRISHTI-GS1 dataset^49^ includes 101 retinal images for glaucoma assessment. The images were captured with a $30^\circ$ FOV centered on the optic disc (OD) and are of size 2896$\times$1944 pixels. Average boundaries of both the optic cup (OC) and the OD in all images were obtained from manual annotations by four experts. The dataset is divided into 50 images for training and 51 for testing. We refer to the OC boundaries as the DRISHTI-OC dataset.

#### DRISHTI-OD

The DRISHTI-OD dataset refers to average boundaries of the OD regions in the 101 retinal images of the DRISHTI-GS1 dataset^49^ described above.

#### PROMISE12

The PROMISE12 (Prostate MR Image Segmentation 2012) dataset^50^ contains three-dimensional (3D) transversal T2-weighted magnetic resonance (MR) images of 50 patients scanned at various centers using various MRI scanners and imaging protocols. The size of the images varies, from 256$\times$256 pixels, to 320$\times$320, 384$\times$384, and 512$\times$512 pixels. In our experiments we used only images of patients 0-12, all of size 512$\times$512 pixels, of which we used 200 for training and 74 for testing^51^.

#### BCSS

The BCSS (Breast Cancer Semantic Segmentation) dataset^52^ contains more than 20,000 manually segmented tissue regions in 151 whole-slide breast-cancer images from The Cancer Genome Atlas (TCGA). The images vary in size, 1500-3000$\times$2000-4000 pixels, and were annotated by 25 participants ranging in experience from senior pathologists to medical students. Following others^53^, we used 100 images for training and the remaining 51 for testing.

### Segmentation performance metrics

To quantify segmentation performance, we used seven popular metrics^54,55^. Denoting the segmented image by *S* and the corresponding ground-truth image by *G*, each having *N* pixels $i=1\dots N$ with a value either 0 (negative $=$ background) or 1 (positive $=$ foreground), we first computed the numbers of true-positive (TP) pixels:

7 $$\begin{aligned} \text {TP} = \sum ^N_{i=1} S_i\cdot G_i, \end{aligned}$$

true-negative (TN) pixels:

8 $$\begin{aligned} \text {TN} = \sum ^N_{i=1} (1-S_i)\cdot (1-G_i), \end{aligned}$$

false-positive (FP) pixels:

9 $$\begin{aligned} \text {FP} = \sum ^N_{i=1} S_i\cdot (1-G_i), \end{aligned}$$

and false-negative (FN) pixels:

10 $$\begin{aligned} \text {FN} = \sum ^N_{i=1} (1-S_i)\cdot G_i, \end{aligned}$$

from which we obtained the sensitivity (Se), also known as the recall (R):

11 $$\begin{aligned} \text {Se} = \text {R} = \frac{\text {TP}}{\text {TP}+\text {FN}}, \end{aligned}$$

the specificity (Sp):

12 $$\begin{aligned} \text {Sp} = \frac{\text {TN}}{\text {TN}+\text {FP}}, \end{aligned}$$

the accuracy (A):

13 $$\begin{aligned} \text {A} = \frac{\text {TP}+\text {TN}}{\text {TP}+\text {TN}+\text {FP}+\text {FN}}, \end{aligned}$$

the balance accuracy (BA):

14 $$\begin{aligned} \text {BA} = \frac{\text {Se}+\text {Sp}}{2}, \end{aligned}$$

the Dice (D) coefficient, which is equivalent to the F1-score:

15 $$\begin{aligned} \text {D} = \text {F1} = \frac{2|S\cap G|}{|S|+|G|} = \frac{2\text {TP}}{2\text {TP}+\text {FP}+\text {FN}}, \end{aligned}$$

the Jaccard (J) coefficient:

16 $$\begin{aligned} \text {J} = \frac{|S\cap G|}{|S\cup G|} = \frac{\text {TP}}{\text {TP}+\text {FP}+\text {FN}}, \end{aligned}$$

and the overlap error (E):

17 $$\begin{aligned} \text {E} = 1 - \text {J} = \frac{\text {FP}+\text {FN}}{\text {TP}+\text {FP}+\text {FN}}. \end{aligned}$$

The values of all metrics are in the range [0, 1], where 0 means worst and 1 means best performance, except for E, where 0 means best and 1 means worst performance.

**Table 3:** Effect of input image downsampling on segmentation performance for the considered datasets.

Downsampling	Se	Sp	A	BA	D	J	E	DE	MNF	MDF	PC
	**STARE**							0.2105	0.3725	0.1796	0.1971
2	0.8741	0.9915	0.9826	0.9328	0.8833	0.7913	0.2087				
3	0.8086	0.9872	0.9738	0.8979	0.8226	0.6991	0.3009				
4	0.7586	0.9868	0.9696	0.8727	0.7895	0.6527	0.3473				
	**DRIVE**							0.2821	0.4632	0.2301	0.2253
2	0.8077	0.9872	0.9715	0.8975	0.8317	0.7121	0.2879				
3	0.6931	0.9799	0.9549	0.8365	0.7282	0.5731	0.4269				
4	0.6242	0.9767	0.9460	0.8005	0.6683	0.5027	0.4973				
	**CHASE-DB1**							0.1869	0.3961	0.1967	0.2670
2	0.8785	0.9911	0.9837	0.9355	0.8755	0.7788	0.2212				
3	0.8280	0.9872	0.9769	0.9076	0.8233	0.7002	0.2998				
4	0.7655	0.9866	0.9723	0.8761	0.7818	0.6427	0.3573				
	**MC**							0.0594	0.0367	0.0166	0.0016
2	0.9996	0.9997	0.9997	0.9996	0.9993	0.9986	0.0014				
3	0.9991	0.9996	0.9995	0.9991	0.9990	0.9980	0.0020				
4	0.9990	0.9995	0.9994	0.9990	0.9987	0.9975	0.0025				
	**PH2**							0.0248	0.0181	0.0049	0.0014
2	0.9985	0.9975	0.9984	0.9985	0.9965	0.9931	0.0069				
3	0.9966	0.9974	0.9980	0.9966	0.9955	0.9910	0.0090				
4	0.9958	0.9962	0.9971	0.9958	0.9936	0.9873	0.0127				
	**ISIC-2016**							0.0093	0.0106	0.0017	0.0128
2	0.9979	0.9994	0.9995	0.9979	0.9976	0.9953	0.0047				
3	0.9968	0.9990	0.9992	0.9968	0.9962	0.9925	0.0075				
4	0.9964	0.9990	0.9991	0.9964	0.9961	0.9922	0.0078				
	**DRISHTI-OC**							0.0090	0.0128	0.0072	0.0029
2	0.9943	1.0000	0.9990	0.9971	0.9961	0.9922	0.0078				
3	0.9943	0.9999	0.9998	0.9971	0.9950	0.9901	0.0099				
4	0.9901	0.9999	0.9997	0.9950	0.9918	0.9838	0.0162				
	**DRISHTI-OD**							0.0117	0.0104	0.0045	0.0013
2	0.9957	1.0000	0.9998	0.9978	0.9972	0.9943	0.0057				
3	0.9955	0.9999	0.9998	0.9977	0.9963	0.9925	0.0075				
4	0.9924	0.9998	0.9996	0.9961	0.9939	0.9880	0.0120				
	**PROMISE12**							0.1104	0.0469	0.0175	0.0035
2	0.9623	0.9988	0.9978	0.9805	0.9654	0.9336	0.0664				
3	0.9453	0.9988	0.9969	0.9722	0.9568	0.9178	0.0822				
4	0.9398	0.9985	0.9963	0.9692	0.9499	0.9054	0.0946				
	**BCSS**							0.0282	0.0163	0.0018	0.0085
2	0.9950	0.9988	0.9977	0.9969	0.9963	0.9927	0.0073				
3	0.9944	0.9971	0.9966	0.9957	0.9946	0.9894	0.0106				
4	0.9914	0.9964	0.9953	0.9939	0.9924	0.9851	0.0149				

The proposed complexity measures computed on the original images are also reported.

### Regression analysis performance metrics

To evaluate the performance of the linear regression models, we used the most common regression performance metrics, including the coefficient of determination $\text {R}^2$, adjusted $\text {R}^2$, RMSE, MAE, and important unbiased metrics, namely AIC and its corrected version AICc^56^.

The first is a statistical measure of proportional variance in the outcome that is explained by the independent variables^57^ and is computed as:

18 $${\text{R}}^{2} = 1 - \frac{{{\text{RSS}}}}{{{\text{TSS}}}}$$

with the total sum of squares (TSS)

19 $$\begin{aligned} \text {TSS} = \sum _{i=1}^n \left( y_i - \bar{y} \right) ^2 \end{aligned}$$

and the residual sum of squares (RSS)

20 $$\begin{aligned} \text {RSS} = \sum _{i=1}^n \left( y_i - m_i \right) ^2 \end{aligned}$$

computed from the observed values $y_i$ and the values $m_i$ predicted by the model^57^. The regression model having a higher $\text {R}^2$ value is considered to be better. To account for the numbers of independent variables, *k*, and observations, *n*, the adjusted $\text {R}^2$ ($\text {AR}^2$) is also employed^58^:

21 $$\begin{aligned} \text {AR}^2 = 1-\frac{\left( 1-R^2 \right) \left( n-1 \right) }{\left( n-k-1 \right) }. \end{aligned}$$

To measure the average error of the models in predicting the observations, we computed the RMSE, defined as:

22 $$\begin{aligned} \text {RMSE} = \sqrt{\frac{1}{n}\sum _{i=1}^{n}\left( y_i-m_i \right) ^2}, \end{aligned}$$

as well as the MAE, defined as:

23 $$\begin{aligned} \text {MAE} = \frac{1}{n}\sum _{i=1}^{n}\left| y_i-m_i \right| . \end{aligned}$$

Finally, to get an unbiased estimate of a model’s performance, we computed the AIC metric:

24 $$\begin{aligned} \text {AIC} = n\,\text {log} \left( \frac{\text {RSS}}{n} \right) +2k, \end{aligned}$$

and because our sample size is relatively small ($n=10$ datasets), we also employed the AICc metric:

25 $$\begin{aligned} \text {AICc} = \text {AIC} + \frac{2k^2+2k}{n-k-1}. \end{aligned}$$

### Experiment I: image complexity as a guide for input downsampling

This experiment was designed to investigate the effect of input downsampling on medical image segmentation performance and how the proposed complexity measures predict the corresponding information loss. We considered three downsampling factors: 2, 3, and 4, which are typically sufficient to reduce the images to a workable size for most networks. For this experiment, we did not employ the networks, as the goal was to study the effect of input downsampling alone. To this end, the binary annotation masks of the images of all considered datasets were downsampled by a given factor, and then upsampled with the same factor to restore their size for comparison with the original masks using the segmentation performance metrics (Section "Segmentation performance metrics"). Bilinear interpolation was employed in our implementation for both downsampling and upsampling. To minimize aliasing artifacts in the reconstructions, we removed all frequency components above the resampling Nyquist frequency using a low-pass filter^59^ before downsampling, and after upsampling we applied optimal thresholding to get binary masks maximizing the Dice/F1-measure^60^. From the results of this experiment (Table 3) we observe two important trends: (1) the segmentation quality is consistently decreasing with increasing downsampling, and (2) this effect is less severe for datasets with relatively low image complexity. These trends clearly support our hypothesis that the proposed complexity measures are indicative of the information loss caused by downsampling and therefore can be employed as a guideline to determine the amount of acceptable downsampling.

**Figure 1:**
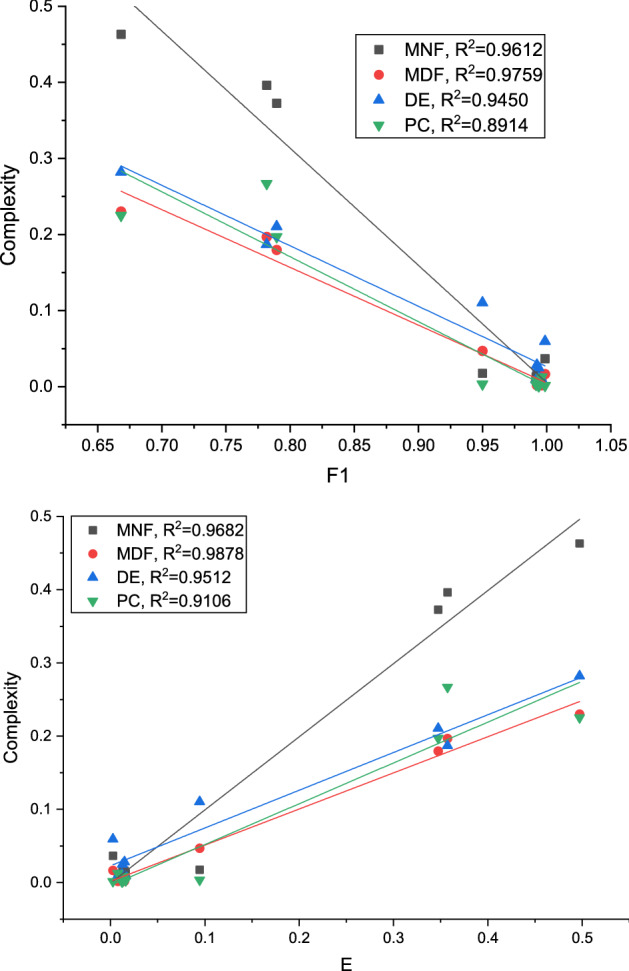
Comparison of complexity measures in terms of their predictive performance.

To compare the predictive power of the different complexity measures on segmentation performance, we performed linear regression for the two most common segmentation performance metrics: Dice (F1) and Jaccard (expressed via E). The results (Fig. 1) indicate that the MDF measure outperforms the other measures in predicting segmentation quality, as confirmed by its highest $\text {R}^2$ values. As both MNF and MDF are higher than DE and PC, it can be concluded that frequency information is most predictive of segmentation performance in the datasets considered in our experiments. The other measures capture different types of complexity and may prove useful in other medical image segmentation tasks.

To evaluate the trade-off between the goodness-of-fit and model complexity in terms of the number of independent variables (or the degree of freedom), we compared the regression performance of models by varying the degree of freedom (DoF) and using the regression performance metrics (Section "Regression analysis performance metrics"). The metrics were computed for the three considered downsampling factors: 2, 3, and 4. The DoF is the number of independent variables in the polynomial function (or the degree of the polynomial) that best fits the data. In our experiments, models with $\text {DoF}>5$ did not improve the regression performance in general (Table 4). More specifically, while performance further improved in terms of the other metrics, according to the AICc metric optimal performance was reached for $\text {DoF}=4$ or 5 in most cases. Given our small sample size, we considered AICc to be decisive owing to its unbiased nature.

**Table 4:** Performance comparison of image complexity measures in terms of regression performance in predicting the error measure E for various downsampling factors and degrees of freedom (DoF) of the regression model based on the results of all datasets. Best values are indicated in bold.

Measure	DoF	${\text {R}^2}$	${\text {AR}^2}$	RMSE	MAE	AIC	AICc
**Downsampled by 2**							
DE	1	0.964387	0.958452	0.021752	0.016354	− 59.2487	− 58.5821
	2	0.964775	0.950684	0.021634	0.016246	− 57.3362	− 54.9362
	3	0.987878	0.978787	0.012691	0.008659	− 63.8702	− 57.8702
	4	0.994052	0.986121	0.008890	0.007013	− 67.5658	− 54.2325
	5	0.999930	0.999754	0.000967	0.000626	− 101.062	**− 71.0617**
	6	**0.999958**	**0.999707**	**0.000746**	**0.000399**	**− 103.223**	− 19.2228
MNF	1	0.992983	0.991814	0.009655	0.008384	− 72.2439	− 71.5772
	2	0.998666	0.998133	0.004209	0.003328	− 83.5268	− 81.1268
	3	0.999446	0.999030	0.002714	0.002272	− 88.5520	− 82.5520
	4	0.999833	0.999610	0.001490	0.001202	− 96.1454	**− 82.8121**
	5	0.999972	0.999903	0.000606	0.000369	− 108.533	− 78.5332
	6	**0.999992**	**0.999942**	**0.000331**	**0.000177**	**− 116.238**	-32.2378
MDF	1	0.993635	0.992574	0.009196	0.007828	− 73.0241	− 72.3574
	2	0.997561	0.996585	0.005693	0.004479	− 78.6963	− 76.2963
	3	0.998331	0.997079	0.004709	0.003594	− 79.7330	− 73.7330
	4	0.999695	0.999288	0.002014	0.001472	− 91.3206	− 77.9873
	5	0.999989	0.999961	0.000382	0.000236	− 115.902	**− 85.9019**
	6	**0.999996**	**0.999971**	**0.000235**	**0.000126**	**− 121.711**	-37.7109
PC	1	0.942353	0.932746	0.027675	0.016837	− 55.3957	− 54.7290
	2	0.966339	0.952875	0.021148	0.015842	− 57.6996	− 55.2996
	3	0.982266	0.968966	0.015350	0.011684	− 60.8266	− 54.8266
	4	0.999788	0.999506	0.001677	0.001038	− 94.2488	**− 80.9155**
	5	0.999806	0.999322	0.001604	0.000961	− 92.9601	− 62.9601
	6	**0.999966**	**0.999763**	**0.000670**	**0.000377**	**− 104.927**	-20.9274
**Downsampled by 3**							
DE	1	0.971305	0.966522	0.028192	0.020556	− 55.0995	− 54.4329
	2	0.974376	0.964127	0.026640	0.019587	− 54.0052	− 51.6052
	3	0.992955	0.987671	0.013969	0.009600	− 62.3344	− 56.3344
	4	0.996215	0.991169	0.010238	0.008100	− 65.3059	− 51.9726
	5	0.999964	0.999875	0.000994	0.000635	− 100.623	**− 70.6233**
	6	**0.999989**	**0.999924**	**0.000548**	**0.000293**	**− 108.159**	-24.1591
MNF	1	0.985851	0.983493	0.019796	0.015826	− 60.7561	-60.0894
	2	0.996766	0.995473	0.009464	0.006754	− 70.5639	− 68.1639
	3	0.996932	0.994631	0.009218	0.006069	− 68.9855	− 62.9855
	4	0.999801	0.999537	0.002345	0.001862	− 88.8881	− 75.5548
	5	0.999991	0.999970	0.000486	0.000303	− 112.080	**− 82.0802**
	6	**1.000000**	**0.999999**	**0.000008**	**0.000004**	**− 139.762**	− 55.7619
MDF	1	0.986262	0.983972	0.019507	0.015018	− 60.9920	− 60.3254
	2	0.994309	0.992032	0.012555	0.009130	− 66.0420	− 63.6420
	3	0.994451	0.990290	0.012397	0.008372	− 64.2447	− 58.2447
	4	0.999674	0.999240	0.003003	0.002189	− 84.9303	− 71.5970
	5	0.999988	0.999958	0.000574	0.000346	− 109.406	**− 79.4060**
	6	**0.999996**	**0.999975**	**0.000314**	**0.000169**	**− 117.049**	− 33.0493
PC	1	0.918892	0.905374	0.047397	0.027916	− 46.7871	− 46.1204
	2	0.954481	0.936273	0.035507	0.026147	− 49.4083	− 47.0083
	3	0.974561	0.955482	0.026544	0.019937	− 52.0630	− 46.0630
	4	0.999846	0.999641	0.002064	0.001275	− 90.9320	**− 77.5987**
	5	0.999852	0.999480	0.002028	0.001215	− 89.2110	− 59.2110
	6	**0.999974**	**0.999819**	**0.000847**	**0.000476**	**− 101.187**	− 17.1869
**Downsampled by 4**							
DE	1	0.968211	0.962913	0.034523	0.025492	− 51.8580	− 51.1913
	2	0.970937	0.959316	0.033010	0.024596	− 50.5752	− 48.1752
	3	0.990215	0.982877	0.019153	0.013639	− 57.2843	− 51.2843
	4	0.994972	0.988268	0.013730	0.010878	− 60.6105	− 47.2772
	5	0.999867	0.999536	0.002231	0.001323	**− 87.6878**	**− 57.6878**
	6	**0.999885**	**0.999193**	**0.002079**	**0.001114**	− 86.8103	− 2.81032
MNF	1	0.986812	0.984614	0.022236	0.018458	− 58.8966	− 58.2299
	2	0.997850	0.996990	0.008978	0.006973	− 71.4084	− 69.0084
	3	0.998171	0.996799	0.008281	0.006226	− 70.7005	− 64.7005
	4	0.999691	0.999278	0.003406	0.002665	− 82.9140	− 69.5806
	5	0.999909	0.999682	0.001847	0.001183	− 90.7113	**− 60.7113**
	6	**0.999958**	**0.999709**	**0.001248**	**0.000668**	**− 94.9813**	− 10.9813
MDF	1	0.987747	0.985705	0.021433	0.017538	− 59.4849	− 58.8182
	2	0.995947	0.994325	0.012328	0.009407	− 66.3344	− 63.9344
	3	0.996180	0.993315	0.011968	0.008978	− 64.8085	− 58.8085
	4	0.999414	0.998633	0.004686	0.003487	− 77.8094	− 64.4761
	5	0.999990	0.999964	0.000622	0.000438	− 108.118	− 78.1177
	6	**1.000000**	**1.000000**	**6.54E-06**	**3.52E-06**	**− 179.008**	**− 95.0082**
PC	1	0.924150	0.911508	0.053327	0.032039	− 44.9009	− 44.2342
	2	0.954159	0.935823	0.041457	0.030712	− 46.9295	− 44.5295
	3	0.974786	0.955876	0.030746	0.023560	− 49.7118	− 43.7118
	4	0.999678	0.999250	0.003473	0.002166	− 82.6055	**− 69.2721**
	5	0.999721	0.999023	0.003235	0.001967	− 81.7402	− 51.7402
	6	**0.999977**	**0.999836**	**0.000937**	**0.000526**	**− 99.5720**	− 15.5720

**Table 5:** Effect of input image downsampling on the segmentation performance of U-Net compared to no downsampling for selected high- and low-complexity datasets.

Image size	Se	Sp	A	BA	D	J	E	%FG	%BG
**DRIVE** ($\text {MDF}=0.2301$)									
Original	0.8312	0.9828	0.9693	0.9069	0.8257	0.7036	0.2964	8.6947	91.3053
Downsampled by 2	0.8018	0.9801	0.9551	0.8910	0.8011	0.6521	0.3479	8.6650	91.3350
Downsampled by 3	0.7541	0.9774	0.9579	0.8658	0.7576	0.6102	0.3898	8.6451	91.3549
Downsampled by 4	0.7293	0.9758	0.9542	0.8526	0.7357	0.5822	0.4178	8.6278	91.3722
**CHASE-DB1** ($\text {MDF}=0.1967$)									
Original	0.8289	0.9848	0.9739	0.9069	0.8179	0.6915	0.3085	7.3391	92.6609
Downsampled by 2	0.8119	0.9829	0.9714	0.8974	0.7995	0.6698	0.3302	7.3991	92.6009
Downsampled by 3	0.7977	0.9821	0.9671	0.8899	0.7731	0.6391	0.3609	7.4457	92.5543
Downsampled by 4	0.7809	0.9801	0.9667	0.8846	0.7569	0.6219	0.3781	7.5866	92.4134
**DRISHTI-OC** ($\text {MDF}=0.0072$)									
Original	0.9449	0.9990	0.9971	0.9803	0.9117	0.8441	0.1559	1.7148	98.2852
Downsampled by 2	0.9446	0.9980	0.9968	0.9798	0.9113	0.8433	0.1567	1.7139	98.2861
Downsampled by 3	0.9411	0.9989	0.9965	0.9788	0.9098	0.8392	0.1608	1.7119	98.2881
Downsampled by 4	0.9344	0.9977	0.9960	0.9760	0.9018	0.8359	0.1641	1.7122	98.2878
**DRISHTI-OD** ($\text {MDF}=0.0045$)									
Original	0.9681	0.9990	0.9980	0.9836	0.9560	0.9207	0.0793	3.1343	96.8657
Downsampled by 2	0.9679	0.9980	0.9970	0.9830	0.9558	0.9202	0.0798	3.1323	96.8677
Downsampled by 3	0.9673	0.9980	0.9970	0.9827	0.9557	0.9198	0.0802	3.1290	96.8710
Downsampled by 4	0.9669	0.9980	0.9970	0.9825	0.9555	0.9195	0.0805	3.1289	96.8711

**Figure 2:**
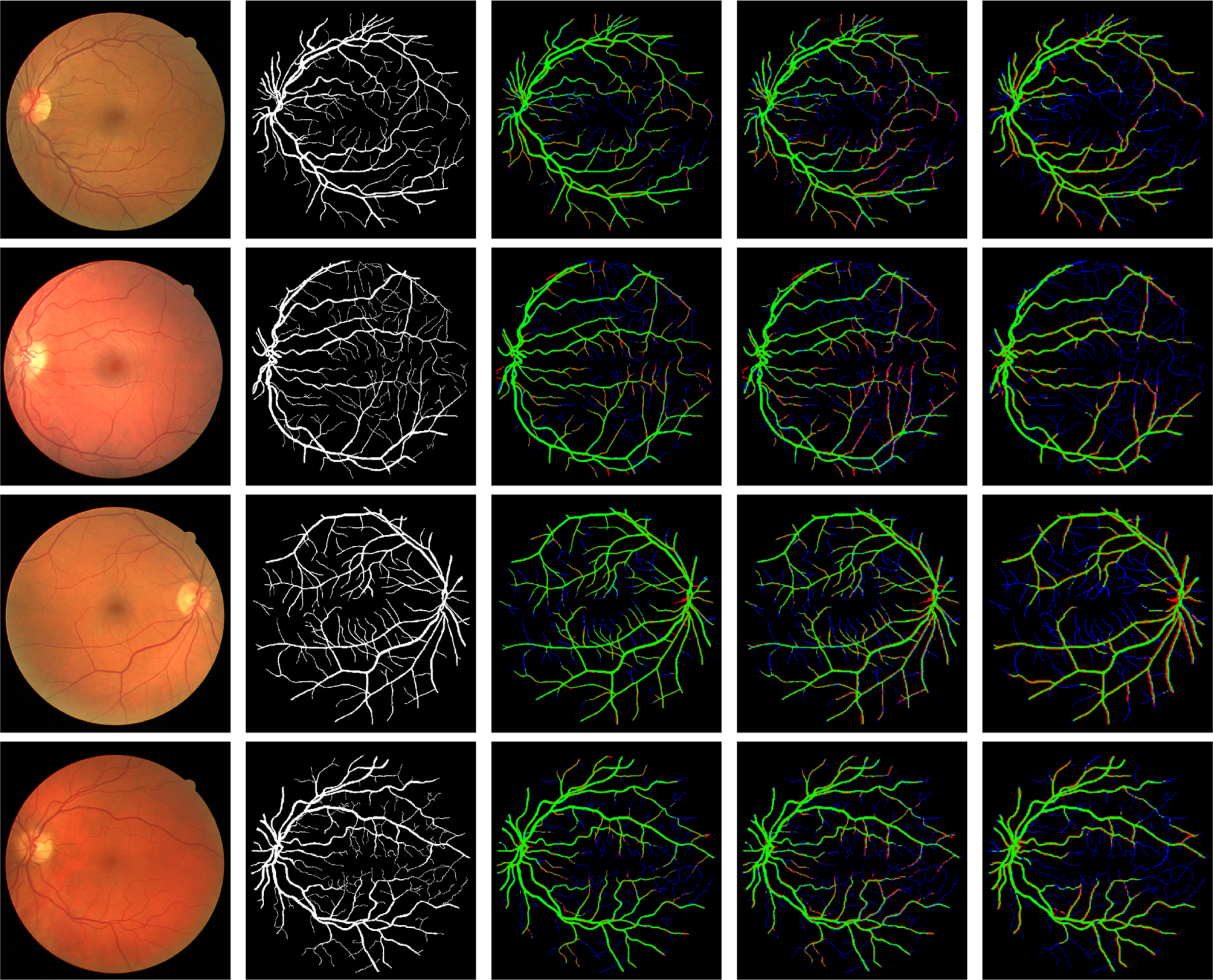
Sample segmentation results with U-Net on the DRIVE dataset. Four examples are shown from top to bottom. From left to right: the input images, the ground truth manually annotated by an expert, and the results on $2\times$, $3\times$, and $4\times$ downsampled input images. Correctly segmented foreground and background pixels are shown in, respectively, green and black. False positive and false negative pixels are shown in, respectively, red and blue.

**Figure 3:**
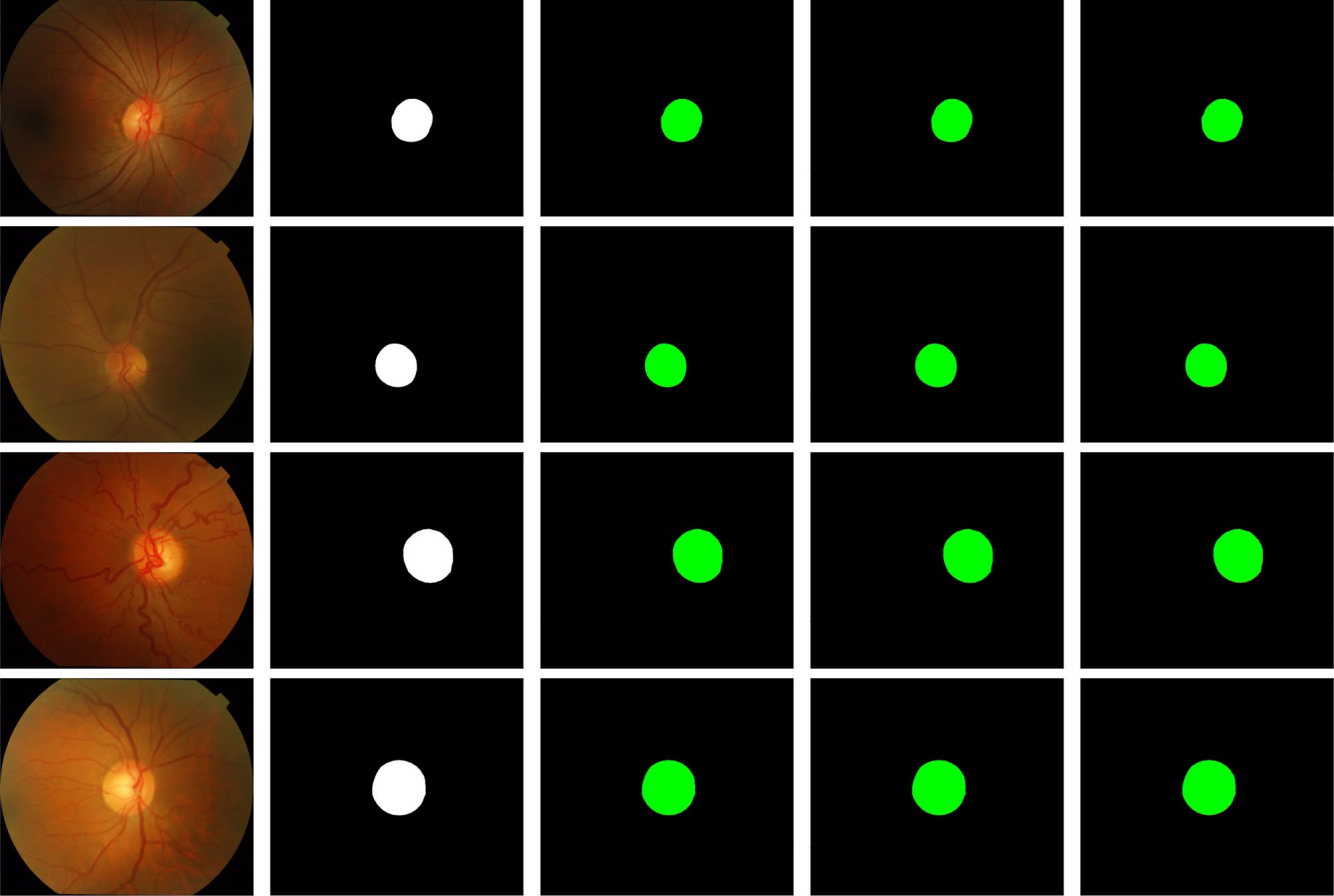
Sample segmentation results with U-Net on the DRISHTI-OC dataset. Four examples are shown from top to bottom. From left to right: the input images, the ground truth manually annotated by an expert, and the results on $2\times$, $3\times$, and $4\times$ downsampled input images. Correctly segmented foreground and background pixels are shown in, respectively, green and black. False positive and false negative pixels are shown in, respectively, red and blue (visible around the object edges only at very high magnification).

**Figure 4:**
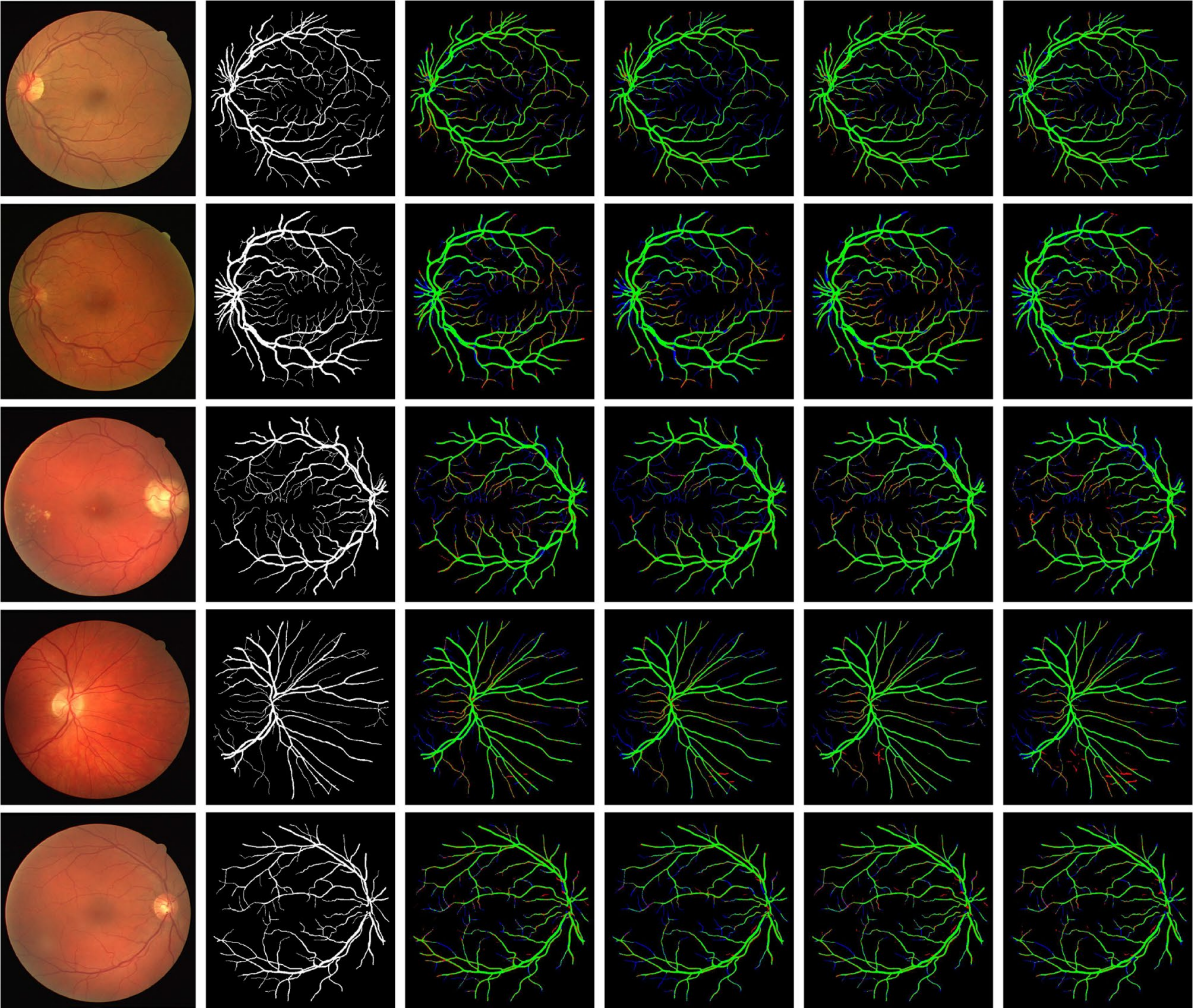
Sample segmentation results of the four different networks on the DRIVE dataset. Five examples are shown from top to bottom. From left to right: the input images, the ground truth manually annotated by an expert, and the results on DeeplabV3+, M2U-Net, U-Net, and U-Net Lite. Correctly segmented foreground and background pixels are shown in, respectively, green and black. False positive and false negative pixels are shown in, respectively, red and blue.

**Figure 5:**
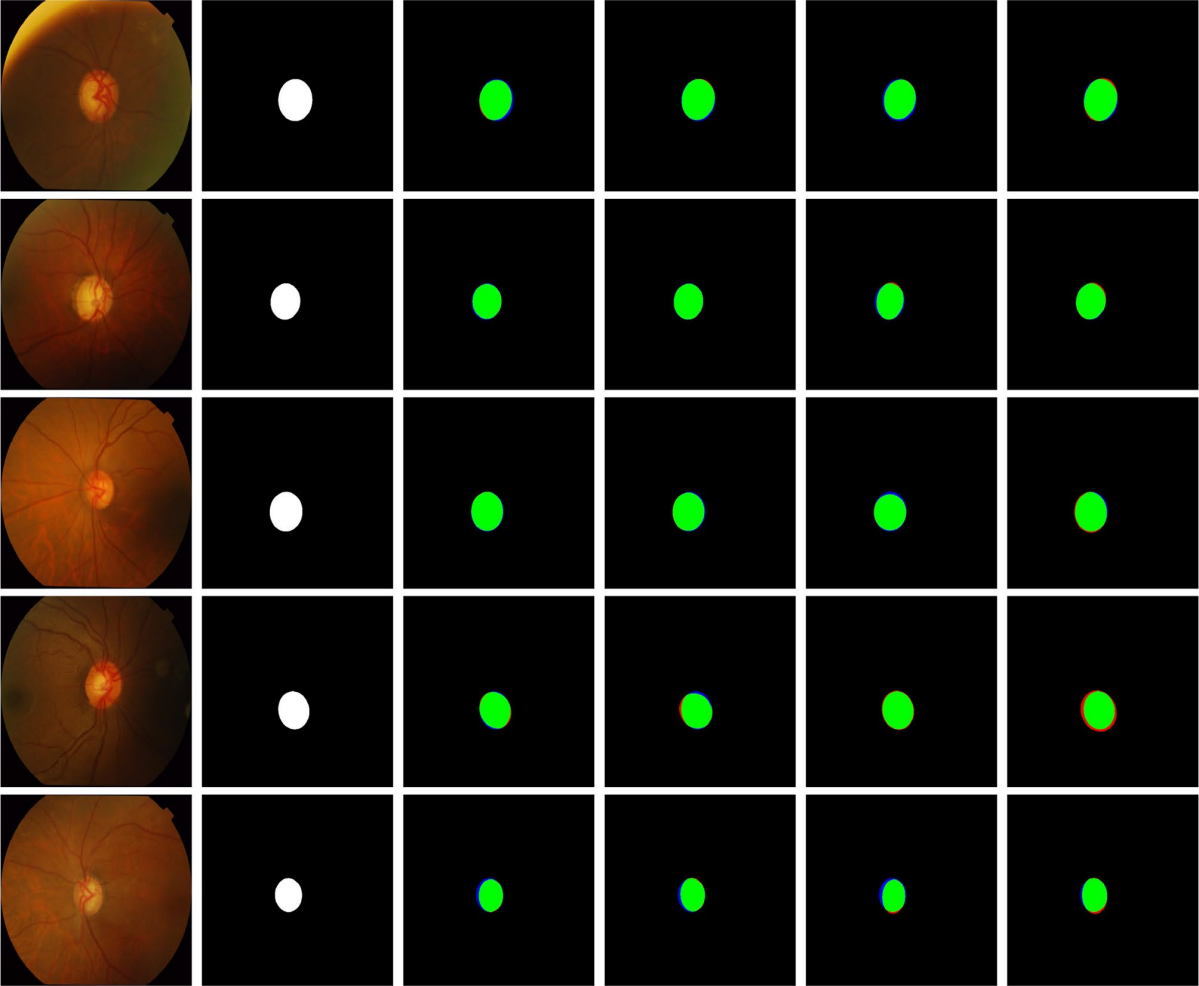
Sample segmentation results of the four different networks on the DRISHTI-OC dataset. Five examples are shown from top to bottom. From left to right: the input images, the ground truth manually annotated by an expert, and the results on DeeplabV3+, M2U-Net, U-Net and U-Net Lite. Correctly segmented foreground and background pixels are shown in, respectively, green and black. False positive and false negative pixels are shown in, respectively, red and blue (visible around the object edges only at very high magnification).

**Figure 6:**
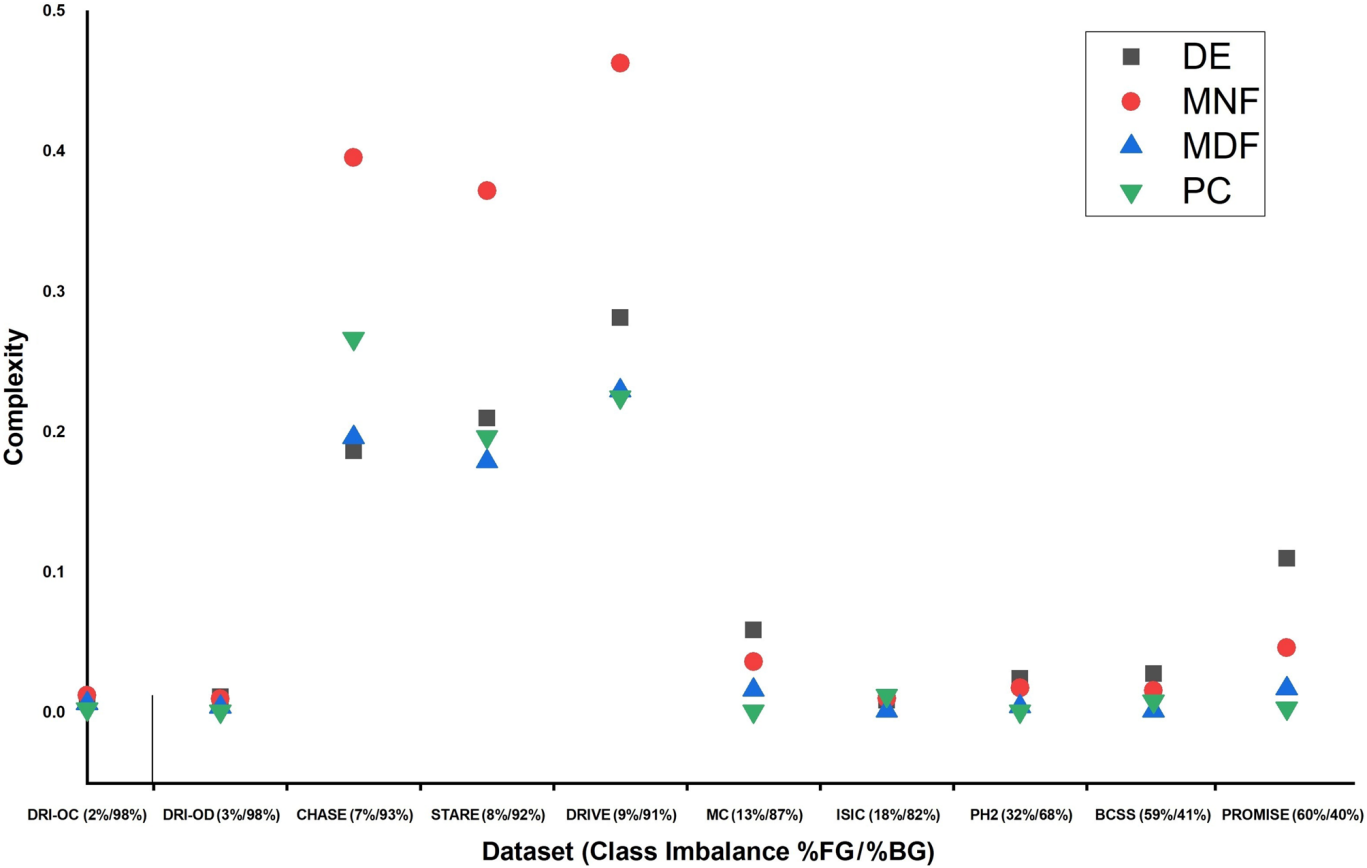
Effect of class imbalance on complexity measures.

To reaffirm the predictive power of the proposed image complexity measures for segmentation performance, we trained U-Net (Section "Segmentation networks") with the original images and separately with downsampled images (factors 2, 3, 4) from two relatively high-complexity datasets (DRIVE and CHASE-DB1) and two relatively low-complexity datasets (DRISHTI-OC and DRISHTI-OD). From the quantitative results (Table 5) we again observe that segmentation performance consistently decreases with increasing downsampling factor, and the loss is more pronounced for the high-complexity datasets. For example, in this experiment the performance loss was 17% in J, with an increase of 41% in E, for a downsampling factor of 4 on the DRIVE dataset. Similarly, a decrease of 9% in J and an increase of 23% in E was seen in the CHASE-DB1 dataset for the same downsampling factor. By contrast, as expected, no noteworthy loss in segmentation performance was observed in either of the DRISHTI datasets, due to their low complexity. This is confirmed by visual inspection (Figs. 2 and 3). We also notice that with increasing downsampling, the number of false negatives increased more than the number of false positives in the DRIVE dataset. This was to be expected, as it is increasingly harder for the deep networks to capture the tiny vessels, which tend to get lost in the downsampling process. In the DRISHTI dataset, on the other hand, the loss due to downsampling is negligible. Further segmentation results for the DRIVE dataset (Fig. 4) and DRISHTI-OC dataset (Fig. 5) illustrate the performance of the four different networks. The percentages of foreground (FG) and background (BG) pixels (Table 5), which represent the class imbalance in the datasets, are not affected by image downsampling, as expected. Plotting the class imbalance of the datasets against the proposed complexity measures showed no direct relationship between these variables (Fig. 6).

**Figure 7:**
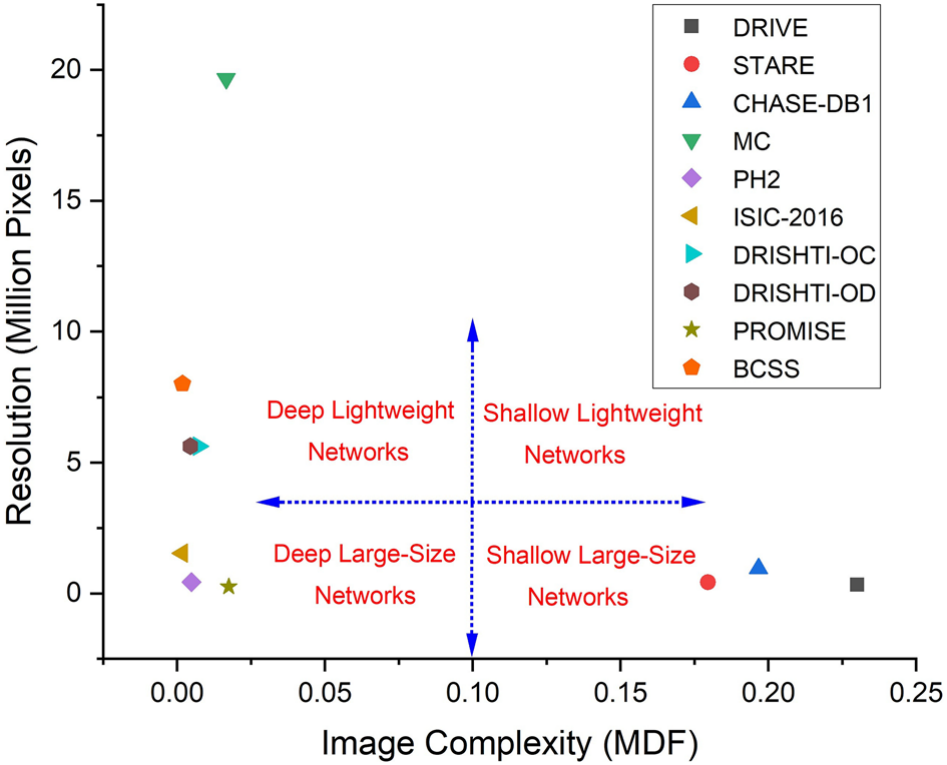
Framework for designing medical image segmentation networks. Macro-level network design choices are depicted in red. The ranges are indicative based on our experiments and are subject to the task at hand.

### Experiment II: network selection based on image complexity

In this experiment, we investigated the suitability of image complexity as a guideline in choosing a deep large-size, deep lightweight, shallow large-size, or shallow lightweight network for segmentation. The assumption here was that training a deep network on moderate hardware would necessitate downsampling of the input images. To evaluate the impact of this, we used the DRIVE dataset, which has high image complexity, and a combination of datasets, ISIC-2016 (training set) and PH2 (test set), which have low complexity. Since we learned from the previous experiment (Table 5) that performance on the DRIVE dataset decreases as the amount of downsampling increases, in the second experiment we examined the impact of formidable downsampling (factor 4) on both high and low-complexity sets on the performance of the considered networks.

The experimental results (Table 6) show that when image complexity is high, downsampling by 4 has a negative impact on the performance of all four networks. For example, for DeepLabV3+, the J for the downsampled data was about 18% lower than the original data, and E about 36% higher. We can see that on the high-complexity dataset DRIVE, the shallow large-size U-Net performed better than the other three networks. The shallow lightweight U-Net Lite, which has nearly 100 times fewer parameters than the U-Net, performed well too. Thus, we can conclude that shallow networks are best suited for high-complexity datasets in general. For high-resolution, high-complexity datasets, a shallow lightweight network is most practical, as it is computationally faster.

We also observe that when image complexity is low, each of the four networks performed comparably on the original and the downsampled images (Table 6). For example, for DeepLabV3+, the J for the downsampled data was only about 1% lower than the original data, and E only about 5% higher. Overall, this network performed better than the other three, and the deep lightweight M2U-Net performed better than the two shallow networks. The J for M2U-Net was only about 3% lower than for DeepLabV3+, and E around 15% higher, while the former network has 36 times fewer trainable parameters. Our results advocate the choice of deep networks for low-complexity datasets. Moreover, a deep lightweight alternative achieves competitive performance when dealing with high-resolution, low-complexity datasets, but at considerably lower computational cost.

**Table 6:** Performance of DeepLabV3+, M2U-Net, U-Net, and U-Net Lite on the high-complexity DRIVE dataset and the low-complexity ISIC-2016/PH2 dataset.

Network	Se	Sp	A	BA	D	J	E	Layers	Parameters
**DRIVE** ($\text {MDF}=0.2301$)									
DeepLabV3+	0.8157	0.9798	0.9654	0.8978	0.8048	0.6737	0.3263	100	20M
	0.6946	0.9463	0.9243	0.8205	0.6552	0.5556	0.4444		
M2U-Net	0.8229	0.9826	0.9630	0.9028	0.8091	0.6960	0.3040	155	0.55M
	0.7275	0.9782	0.9571	0.8579	0.7505	0.6009	0.3991		
U-Net	0.8312	0.9828	0.9693	0.9069	0.8257	0.7036	0.2964	58	30M
	0.7552	0.9759	0.9566	0.8656	0.7525	0.6036	0.3956		
U-Net Lite	0.8144	0.9826	0.9678	0.8985	0.8179	0.6984	0.3016	46	0.28M
	0.7248	0.9778	0.9558	0.8513	0.7409	0.5889	0.4111		
**Trained on ISIC-2016 / Tested on PH2** ($\text {MDF}=0.0017 / 0.0049$)									
DeepLabV3+	0.8996	0.9096	0.9468	0.9026	0.9026	0.8290	0.1710	100	20M
	0.8911	0.9059	0.9398	0.9026	0.8985	0.8210	0.1790		
M2U-Net	0.9089	0.9046	0.9339	0.9048	0.8887	0.8015	0.1985	155	0.55M
	0.9130	0.8799	0.9279	0.8965	0.8774	0.7982	0.2018		
U-Net	0.9136	0.8799	0.9297	0.8907	0.8750	0.7966	0.2034	58	30M
	0.8820	0.8711	0.9290	0.8766	0.8723	0.7904	0.2096		
UNet Lite	0.8898	0.8756	0.9247	0.8611	0.8611	0.7803	0.2197	46	0.28M
	0.8891	0.8732	0.9233	0.8612	0.8607	0.7783	0.2217		

For each dataset and network, two rows of performance values are given, where the top values are the performances at normal image resolution and the bottom values are the performances when the images are downsampled by a factor of 4. The number of layers and parameters (millions) of each network are also listed for reference.

### Network design framework for medical image segmentation

Networks for medical image segmentation often have a large number of model parameters and require multi-GPU compute resources for training. Leaderboard methods in polyp, retinal vessel, and skin lesion segmentation benchmarks are a few representative examples^45,61,62^. Image downsampling is common in applying these methods in order to offset the computational load during training^20,61^. Lightweight approaches for medical and generic image segmentation targeted at embedded platforms either predetermine the architectural choices^28^ or iteratively search for topologies to minimize some objective^13^. Common to all these approaches is dataset (task) independent network design. In this work, we recommend that the complexity of the dataset be an important factor in macro-level network design, specifically the depth of the network and the number of feature channels per layer.

Based on our experiments, we put forward a generic framework for designing neural networks for medical image segmentation (Fig. 7). The macro-level design choices include the number of layers in the network (deep versus shallow) and the representational power within each layer (large-size versus lightweight). Depending on the complexity and resolution of the dataset, one of the four macro-level design combinations can be adopted for network design. We note that image complexity guides the choice between deep and shallow networks, whereas the resolution is important in deciding between lightweight and large-size networks. Categorically, for high-complexity datasets, shallow architectures are a fitting choice, whereas deep networks are more appropriate for low-complexity datasets. We demonstrate the efficacy of the proposed framework by mapping ten benchmark medical datasets to network design choices based on their complexity and resolution. These mappings are supported by the quantitative and qualitative results of Experiment II (Section 4.6). Our complexity-based framework can be employed to guide network design for any new medical image segmentation benchmark or challenge.

## Conclusion

Based on image complexity measures, we presented a framework to guide developers in making several critical macro-level neural network design choices for medical image segmentation. The proposed framework is independent of the segmentation task at hand and the image modalities used. This is possible because the design choices are based solely upon the information contained in the dataset. Extensive experiments on 10 different medical image segmentation benchmarks demonstrated the suitability of our framework. We conclude that the proposed image complexity measures help address the following critical issues in designing a neural network for medical image segmentation: (1) design and train neural networks for high-resolution medical images using generally available moderate computing resources, (2) minimizing the effects of downsampling the input images (usually to aid training) on segmentation performance, and (3) deciding on the depth and size of the architecture (number of layers/parameters) for a given medical image segmentation task. We suggest that our framework complements NAS approaches and can be employed at the macro-level stage in conjunction with NAS for micro-level architectural optimization. In future work we aim to test this hypothesis and perform more extensive experiments on a wider range of different neural network architectures for medical image segmentation as well as other applications.

**Table 7:** URLs of public datasets used in the experiments.

Dataset	URL
STARE	https://cecas.clemson.edu/~ahoover/stare/
DRIVE	https://drive.grand-challenge.org/
CHASE-DB1	https://blogs.kingston.ac.uk/retinal/chasedb1/
MC	https://lhncbc.nlm.nih.gov/LHC-publications/pubs/TuberculosisChestXrayImageDataSets.html
PH2	https://www.fc.up.pt/addi/ph2database.html
ISIC-2016	https://challenge.isic-archive.com/landing/2016
DRISHTI-OC	https://cvit.iiit.ac.in/projects/mip/drishti-gs/mip-dataset2/Dataset.php
DRISHTI-OD	https://cvit.iiit.ac.in/projects/mip/drishti-gs/mip-dataset2/Dataset.php
PROMISE12	http://promise12.grand-challenge.org
BCSS	https://github.com/PathologyDataScience/BCSS

## Data Availability

The datasets analyzed for this study are accessible via the URLs listed in the URL column of Table 7.
